# Potential Activity Mechanisms of *Aesculus hippocastanum* Bark: Antioxidant Effects in Chemical and Biological In Vitro Models

**DOI:** 10.3390/antiox10070995

**Published:** 2021-06-22

**Authors:** Aleksandra Owczarek, Joanna Kolodziejczyk-Czepas, Joanna Woźniak-Serwata, Anna Magiera, Natalia Kobiela, Katarzyna Wąsowicz, Monika Anna Olszewska

**Affiliations:** 1 Department of Pharmacognosy, Faculty of Pharmacy, Medical University of Lodz, 90-151 Lodz, Poland; anna.magiera@umed.lodz.pl (A.M.); nataliakobiela1995@gmail.com (N.K.); katarzyna.wasowicz6@gmail.com (K.W.); monika.olszewska@umed.lodz.pl (M.A.O.); 2 Department of General Biochemistry, Faculty of Biology and Environmental Protection, University of Lodz, 90-236 Lodz, Poland; joanna.kolodziejczyk@biol.uni.lodz.pl (J.K.-C.); joannawozniak94@gmail.com (J.W.-S.)

**Keywords:** horse-chestnut, esculin, fraxin, (‒)-epicatechin, procyanidin A2, human plasma, hemostasis, peroxynitrite, chronic venous insufficiency

## Abstract

The bark of *Aesculus hippocastanum* is an herbal remedy used in conditions connected with vascular insufficiency; however, there is a lack of data concerning its mechanisms of action. The present work is a preliminary investigation into some of the potential directions of the bark activity. The phytochemically (qualitative UHPLC-PDA-MS/MS and quantitative UHPLC-PDA assays) characterized extract and its four main constituents (esculin, fraxin, (‒)-epicatechin and procyanidin A2) were first evaluated in terms of their antioxidant capacity. All analytes demonstrated dose-dependent scavenging potential towards the most common in vivo oxidants, with particularly advantageous capacity of the extract and its flavan-3-ol constituents against peroxynitrite (3.37–13.26 mmol AA/g), hydroxyl radical (5.03–8.91 mmol AA/g) and superoxide radical (3.50–5.50 mmol AA/g). Moreover, even at low concentrations (1–5 µg/mL), they protected components of human plasma against oxidative damage inflicted by peroxynitrite, preventing oxidation of plasma protein thiols and diminishing the tyrosine nitration and lipid peroxidation. High efficiency of the analytes was also demonstrated in preventing the peroxynitrite-induced nitrative changes of fibrinogen (up to 80% inhibition for (‒)-epicatechin at 50 µg/mL), an important protein of coagulation cascade. Additionally, the extract and its constituents had, at most, moderate inhibitory activity towards platelet aggregation induced by ADP and only negligible influence on clotting times. The results show that, among the investigated properties, the antioxidant activity might, to the highest extent, be responsible for the bark efficacy in vascular disorders, thus supporting its application in those conditions; they also indicate the directions for future research that would allow for better understanding of the bark activity.

## 1. Introduction

Chestnut bark (*Hippocastani cortex*) is a traditional herbal medicine obtained from young branches of *Aesculus hippocastanum* L. (horse-chestnut), a large deciduous tree native to the Balkan Peninsula and cultivated throughout the temperate zone for ornamental purposes [1]. The plant material is applied, mainly externally (bath infusions, creams, ointments and suppositories), in conditions connected with vascular damage and defective blood clotting, such as venous insufficiency, hemorrhoids, cutaneous capillary fragility, as well as oedemas, small bruises and limited skin and subcutaneous tissue inflammations [2]. The area of application of that plant material is thus similar to that of horse-chestnut seeds, whose activity is based on high content of triterpene saponins, mainly escin. The activity mechanisms of seeds and escin are widely researched and relatively well recognized [3]. On the other hand, the clinical efficacy and indications of the horse-chestnut bark rely mainly on its long-term traditional use and there is still insufficient data demonstrating possible mechanisms of the activity [2]. The scarce knowledge about the contribution of the main ingredients to the overall activity makes it also difficult to establish reasonable parameters for plant material pharmacological quality control, despite the fact that some methods for that purpose are already available [4,5].

Most of the information about the potential mechanisms of the bark comes from the 1980s and early 1990s, indicating some venotonic and anti-inflammatory effects [2,6]. More recent research focuses on the antioxidant capacity of the bark, although only a limited number of models (mostly chemical) has been investigated so far [7,8]. In both studies, the relevance of polyphenols has been implied but no individual constituents have been indicated.

Among the main active ingredients of the bark, the sources list coumarin glycosides (mainly esculin and fraxin), as well as flavan-3-ols [4,5,9]; thus, contrary to the seed, the efficiency of the bark may chiefly be the result of polyphenolic constituents. The high antioxidant potential is a distinguishing characteristic of flavan-3-ols, including procyanidins [3], such as those present in *A. hippocastanum* bark. Their role in protecting vital bio-molecules against oxidative damage has been found important to the activity of many recognized vasoprotective agents, e.g., grape seed extract [10]. Coumarins have also been reported as efficient scavengers of reactive oxygen species (ROS) and their efficiency to hinder the oxidative stress is believed to be at least partially responsible for their other biological properties, such as anti-inflammatory action [11]. On the other hand, coumarins are also often linked with anticoagulant effects [3,12], while some anti-aggregatory influence has been demonstrated for procyanidins [13].

Oxidative stress and hemostasis disorders (e.g., the enhanced reactivity of blood platelets and activation of the blood plasma coagulation cascade) connected with undergoing inflammation, often co-occur with venous insufficiency and might play a role in the etiology of the disease, favor its development and/or exacerbate the symptoms [3,14]. Considering its composition, the horse-chestnut bark may alleviate those conditions, but studies are required to establish its detailed mode of action and indicate the constituents chiefly responsible for its efficacy.

Considering the above premises, this in vitro study aimed to gather new information on the potential mechanisms of horse-chestnut bark. The work focused on the bark antioxidant capacity and impact on hemostasis, as well as on the contribution of the individual constituents to those effects. The polyphenolic composition of the extract was first studied qualitatively and quantitatively (UHPLC-PDA-ESI-MS^2^) to indicate and select the potential activity vectors. In the next step, horse-chestnut bark extract and its main constituents were evaluated in terms of direct scavenging of in vivo relevant ROS, as well as in terms of their protective effects on human blood plasma components under the peroxynitrite-induced oxidative stress conditions. The latter evaluation was also complemented by the analysis of their protective action on the nitrative modifications of fibrinogen, an abundant plasma protein, prone to oxidative modifications leading to changes in its biological functions and clotting ability. The influence of the analytes on hemostasis was also further investigated in terms of the analytes anti-thrombotic potential by assessment of their anti-platelet (anti-aggregatory) effects and anticoagulant potential (impact on blood clotting).

## 2. Materials and Methods

### 2.1. General

The analytical grade methanol for extraction was purchased from Avantor Performance Materials (Gliwice, Poland). HPLC grade solvents (acetonitrile, formic acid, orthophosphoric acid) used for UHPLC and HPLC analyses were obtained from Avantor Performance Materials and Sigma-Aldrich (Seelze, Germany/St. Louis, MO, USA). HPLC grade standards of esculin, fraxin, (‒)-epicatechin, procyanidin A2 and ascorbic acid were purchased from Sigma-Aldrich and Phytolab (Vestenbergsgreuth, Germany). All reagents for scavenging assays, the phosphate buffered saline (PBS) and the OPD substrate for peroxidase were purchased from Sigma-Aldrich. Peroxynitrite (ONOO^−^) was synthesized according to Pryor et al. (1995). A Pierce BCA Protein Assay Kit was obtained from Thermo Scientific (Waltham, MA, USA). All immune reagents for 3-nitrotyrosine (3-NT) detection were purchased from Abcam (Cambridge, UK). Redistilled water was used in all analyses. For chemical tests requiring constant temperature, the samples were incubated in a BD 23 incubator (Binder, Tuttlingen, Germany). Scavenging assays and activity tests in blood plasma models were performed in 96-well microplates using a SPECTROstar Nano microplate reader (BMG LabTech, Ortenberg, Germany).

### 2.2. Plant Material and Preparation of the Extract

Commercial samples of horse-chestnut bark (*Hippocastani cortex*) were bought from a local Polish provider, Flos (Mokrsko, Poland), in 2015. A voucher sample (KFG/HB/AHIP_1501_F) was deposited in the Department of Pharmacognosy, Medical University of Lodz, Poland. Prior to the analyses, the plant material was grounded using an electrical grinder and sieved (0.315 mm).

Sample of plant material (100 g) was extracted exhaustively with methanol (3 × 300 mL) and the combined extracts were evaporated in vacuo and lyophilized (Alpha 1–2/LD Plus freeze dryer, Christ, Osterode am Harz, Germany). The obtained dry extract (21.7 g) was stored at 4 °C until use.

### 2.3. Phytochemical Characterization of the Extract

The extract was assessed qualitatively and quantitatively in terms of its polyphenolic constituents using a validated UHPLC-PDA method and the equipment described earlier [5].

### 2.4. Antioxidant Activity in Chemical Models

The superoxide radical (O_2_^•−^) scavenging capacity was evaluated in a xanthine/xanthine oxidase system with NBT used for detection [15]. The ability to scavenge hydrogen peroxide (H_2_O_2_) was assessed by measuring the decrease in levels of quinoneimine forming in the reaction between H_2_O_2_, 4-aminoantipyrine and phenol, catalyzed by horseradish peroxidase [16]. The hydroxyl radical (HO^•^) quenching was assayed by evaluating amounts of HO^•^ in the presence of salicylic acid [16]. The scavenging activity towards nitric oxide (NO^•^) formed from sodium nitroprusside was measured using 4,5-diaminofluorescein as NO^•^ probe [17]. The ability to scavenge ONOO^−^ was determined by measuring the inhibition of Evans blue dye oxidation [18]. For all assays, SC_50_ values (the concentration of the analyte that decreases the initial amount of the oxidant by 50%) were obtained from concentration-scavenging curves (5–10 calibration points) and expressed in µg/mL with respect to the dry weight of the extract or standard. Ascorbic acid (AA) was used as positive control.

### 2.5. Preparation of Plasma Samples

Blood plasma was obtained from buffy coat units purchased from the Regional Centre of Blood Donation and Blood Treatment in Lodz, Poland. Fresh whole blood for experiments on blood platelets was obtained from healthy volunteers and collected at the Ludwik Rydygier Medical Center in Lodz, Poland. Experiments were conducted in accordance with the Declaration of Helsinki. The study design was verified and approved by the committee on the Ethics of Research at the University of Lodz (12/KBBN-UŁ/I/2017). Blood was collected onto CPD (citric acid (3.27 g/L), sodium citrate (26.3 g/L), monobasic sodium phosphate (2.11 g/L) and dextrose (25.5 g/L) and centrifuged to obtain plasma (3000× *g*, 15 min) or platelet rich plasma (PRP; 250× *g*, 10 min).

### 2.6. Antioxidant Activity in Human Plasma Model

Plasma samples were pre-incubated with the examined extract or standards at final concentrations of 1–50 µg/mL for 5 min at 37 °C and then treated with 150 µM (the FRAP assay) or 100 µM (the remaining experiments on blood plasma) of ONOO^−^. Control samples were prepared with plasma without the extract/standards and/or ONOO^−^. No pro-oxidative effect was found in the experiments with plasma and the extract/standards only (without ONOO^−^). The non-enzymatic antioxidant capacity (NEAC) of plasma was evaluated by measuring its ferric-reducing ability (FRAP), according to Marchelak et al. [19], and the results were expressed in mM of Fe^2+^ equivalents. The 3-NT-containing proteins were detected by competitive ELISA (enzyme-linked immunosorbent assay), according to [20]. The levels of nitrated proteins were expressed in the equivalents of 3-nitrotyrosine-containing fibrinogen (3-NT-Fg) (in nmol/mg of plasma proteins). The concentration of free -SH groups in plasma was measured spectrophotometrically using Ellman’s reagent, according to Bijak et al. [10], and expressed in µmol/mL of plasma. The TBARS levels were determined according to Kolodziejczyk et al. [21] and expressed in µmol TBARS/mL of plasma. In all experiments AA was used as positive control.

### 2.7. Protection against Nitrative Modifications of Human Fibrinogen

Fibrinogen was isolated from human plasma (obtained as described in Section 2.4) by the cold ethanol precipitation technique, as described previously [22]. The fibrinogen concentration was established spectrophotometrically at 280 nm using an extinction coefficient of 1.55 for a 1 mg/mL solution. The obtained fibrinogen was pre-incubated with the examined extract/standards at the final concentrations of 1–50 μg/mL for 15 min at room temperature, then treated with 100 μM of ONOO^−^. The detection of 3-NT-containing fibrinogen was performed in the samples by the competitive ELISA method, as described in Section 2.5. The concentration of nitrated proteins was estimated from the standard curve of the nitrated fibrinogen and expressed as the 3-NT-Fg equivalents (in nanomoles per mg of plasma proteins). AA was used as positive control.

### 2.8. Influence on Plasma Hemostasis Parameters

Platelet aggregation was measured in PRP, using the Chrono-Log 490 aggregometer (CHRONO-LOG, Havertown, PA, USA). PRP samples were pre-incubated with the examined extract/standards at the final concentrations of 1–50 µg/mL for 15 min at 37 °C and transferred into aggregometer cuvettes. Aggregation was induced by ADP (at the final concentration of 10 µM) or collagen (at the final concentration of 2 µg/mL). Control samples were untreated with the examined analytes. Aspirin DL-lysine was used as positive control.

Thrombin (TT), prothrombin (PT) and the activated partial thromboplastin time (aPTT) were measured in fresh blood plasma with the use of a Kselmed K-3002 Optic coagulometer (Kselmed, Grudziądz, Poland), using reagents purchased from Diagon Kft. (Budapest, Hungary). Plasma samples were pre-incubated with the examined extract/standards at the final concentration of 1–50 µg/mL for 15 min at 37 °C. Control samples were untreated with the examined analytes. Argatroban was used as positive control.

### 2.9. Statistical Analysis

The quantitative results were expressed as means ± standard error (SE). Normality of the distribution of the results was verified using the Shapiro–Wilk test and the homogeneity of variances using the Levene’s test. The significance of differences between samples and controls was determined with one-way ANOVA, followed by the post-hoc Dunnett’s test for multiple comparisons. All calculations were performed using the Statistica12Pl software for Windows (StatSoft Inc., Krakow, Poland), with *p* values less than 0.05 regarded as significant.

## 3. Results

### 3.1. Extract Standardization

**Figure 1:**
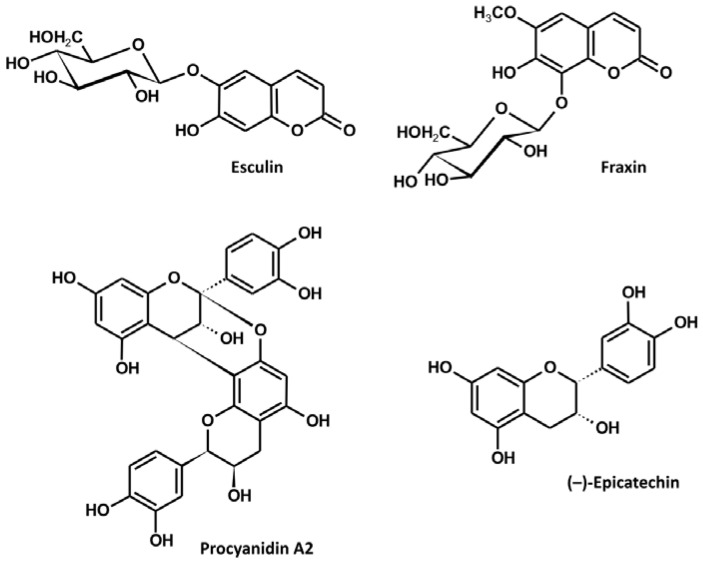
Structures of the major constituents of the *A. hippocastanum* bark.

The investigated extract was assessed qualitatively and quantitatively using the UHPLC-MS-PDA method described previously (Table 1) [5]. Two main groups of compounds detected in the extract were coumarins and proanthocyanidins. In the first group, esculin (over 17% of the extract dry weight) and fraxin (over 7%) prevailed, while, in the latter, (–)-epicatechin (over 6%) and procyanidin A2 (over 5%) were the major constituents (Figure 1). In total, about 40% of the extract could be attributed to simple phenolics detectable by LC-PDA.

### 3.2. Antioxidant Activity in Chemical Models

**Figure 2:**
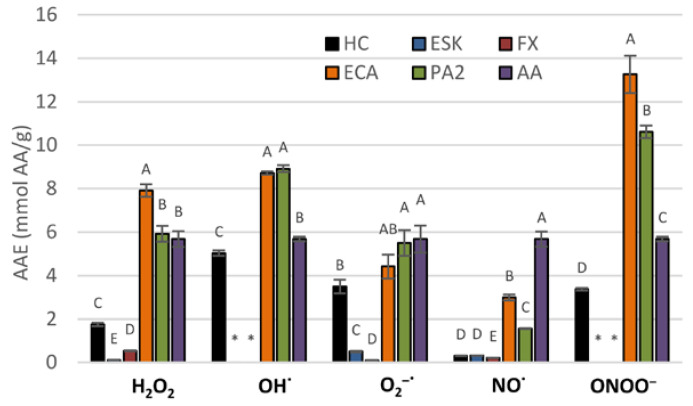
Scavenging activity of the dry methanol extract from the bark of *A. hippocastanum* and its main constituents towards selected reactive oxygen species (ROS), expressed as ascorbic acid equivalents (AAE). Results presented as means ± SE (*n* = 5). For each ROS, the values labeled with the same capital are not statistically different at α = 0.05. Analytes: HC, horse-chestnut bark extract; ESC, esculin; FX, fraxin, ECA, (‒)-epicatechin; PA2, procyanidin A2; AA, ascorbic acid.

The basic antioxidant potential of the extract and its constituents was tested by measuring their scavenging activity towards some of the in vivo operating reactive oxygen species (H_2_O_2_, OH^•^, O_2_^•−^, NO^•^, ONOO^−^). The investigated extract decreased the levels of all of the reactive species in a dose-dependent manner (Table 2, Figure 2). With regard to AA, the extract was most efficient towards OH^•^ with SC_50_ of 172.27 µg/mL, equivalent to 5.03 mmol AA/g; relatively high efficiency was also observed for O_2_^•−^ and ONOO^−^, with SC_50_ of 9.96 µg/mL and 140.44 μg/mL, equivalent to 3.50 mmol AA/g and 3.37 mmol AA/g, respectively. The lowest scavenging potential exhibited by the extract was towards NO^•^, with SC_50_ of 8.74 µg/mL, equivalent to 0.31 mmol AA/g. Among the constituents, flavan-3-ols were highly active. Towards ONOO^−^, H_2_O_2_, O_2_^•−^ and OH^•^, their activity was comparable or higher to that of AA, ranging from 4.41 to 13.26 mmol AA/g. On the other hand, the activity of coumarins was noticeably weaker and did not reach 1 mmol AA/g in any of the experiments.

### 3.3. Antioxidant Activity in Human Plasma Model

**Figure 3:**
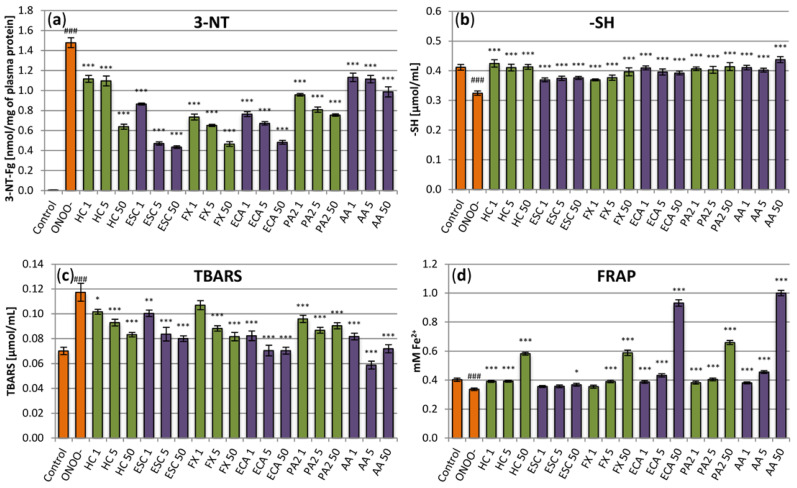
Effects of the investigated analytes on human plasma exposed to oxidative stress. (**a**) Effects on the nitration of plasma proteins assessed by the levels of 3-nirotyrosine (3-NT) and expressed as the 3-nitrotyrosine-containg equivalents (nmol of 3-NT-Fg/mg of plasma proteins); (**b**) effects on plasma protein thiol levels (-SH); (**c**) effects on the non-enzymatic antioxidant capacity (NEAC) of plasma, assessed by ferric reducing ability of plasma (FRAP); (**d**) effects on lipid peroxidation, assessed by the levels of thiobarbituric acid-reactive substances (TBARS). Results presented as means ± SE (*n* = 9). Statistical differences: ### *p* < 0.001 for control plasma versus ONOO^−^-treated plasma (without the investigated analytes); * *p* < 0.05, ** *p* < 0.01, *** *p* < 0.001 for ONOO^−^-treated plasma in the presence of the analytes versus ONOO^−^-treated plasma without the analytes. Analytes: HC, horse-chestnut bark extract; ESC, esculin; FX, fraxin, ECA, (‒)-epicatechin; PA2, procyanidin A2; AA, ascorbic acid; the numbers after analyte symbol indicate concentration in μg/mL.

A more detailed analysis of the antioxidant activity of the extract and its constituents was assessed in the in vitro model of oxidative stress generated in human plasma by ONOO^−^, considered to be one of the most active reactive oxygen species in the cardiovascular system (Figure 3). As strong oxidative/nitrative agent, ONOO^−^ caused measurable and statistically significant (*p* < 0.001) changes in the protein and lipid constituents of the plasma samples. Particularly, it significantly increased the levels of 3-NT in plasma proteins (Figure 3a) and decreased the amount of plasma protein thiols by about 25% (Figure 3b). It also induced peroxidation of lipids, which was evidenced by an increase of about 70% in the levels of TBARS (Figure 3c). Moreover, the exposure to ONOO^−^ weakened the NEAC of plasma, as confirmed by a decline of about 15% in plasma reducing power (Figure 3d). In the samples incubated with the horse-chestnut extract, the changes were partially reduced or, in some cases, effectively prevented (Figure 3). For example, the levels of 3-NT were diminished by about 25–55% and the TBARS levels by about 15–30%, depending on the extract concentration. Moreover, even the smallest tested concentrations of the extract (1 µg/mL and 5 µg/mL) completely prevented the oxidation of plasma protein –SH groups by ONOO^−^ and allowed to maintain the physiological (initial) NEAC of the plasma. On the other hand, the highest concentration (50 µg/mL) improved the NEAC by about 50%. Similar effects were observed for the main extract constituents, although some differences were noticeable in the potency of the individual compounds. (‒)-epicatechin was proved to be the most effective in FRAP and TBARS assays, increasing (at 50 µg/mL) the NEAC by up to 130% and decreasing (at 5–50 µg/mL) TBARS concentration to the base level (control without ONOO^−^). Esculin was the least active compound in FRAP and thiol tests; however, together with fraxin and (‒)-epicatechin, it had an especially pronounced protective impact against tyrosine nitration with the inhibition rate reaching up to 70%.

### 3.4. Influence on the ONOO^−^-Induced Formation of 3-Nitrotyrosine in Fibrinogen

**Figure 4:**
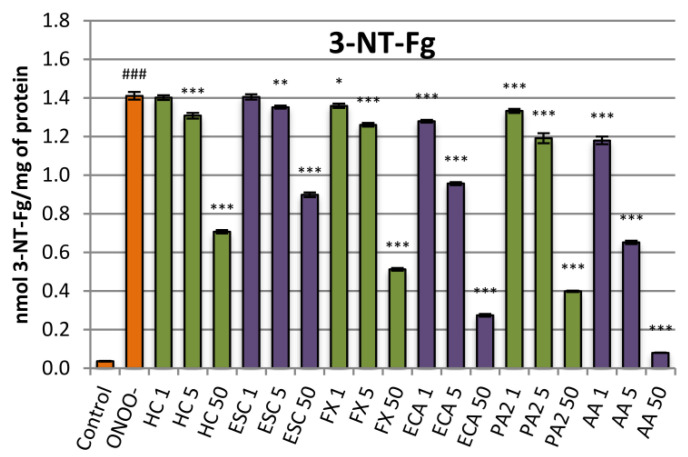
Effects of the investigated analytes on tyrosine nitration in the isolated fibrinogen exposed to ONOO^−^. Results presented as means ± SE (*n* = 10). Statistical differences: ### *p* < 0.001 for control fibrinogen versus ONOO^−^-treated fibrinogen (without the investigated analytes); * *p* < 0.05, ** *p* < 0.01, *** *p* < 0.001 for ONOO^−^-treated fibrinogen in the presence of the analytes versus ONOO^−^-treated fibrinogen without the analytes. Analytes: HC, horse-chestnut bark extract; ESC, esculin; FX, fraxin, ECA, (‒)-epicatechin; PA2, procyanidin A2; AA, ascorbic acid; the numbers after analyte symbol indicate concentration in μg/mL.

The protective effects against nitrative damage of fibrinogen were assessed in the model of the isolated fibrinogen treated with ONOO^−^. Addition of ONOO^−^ to the fibrinogen samples resulted in a significant (*p* < 0.001) increase in the nitrated tyrosine residues (Figure 4). Pre-incubation of the fibrinogen samples with the extract protected the protein against the nitrative damage and reduced the 3-nitrotyrosine content in this protein. The results were statistically significant at concentrations of 5 µg/mL and higher, reaching, at 50 µg/mL, a decrease of about 50% (Figure 4). Among the constituents, the flavan-3-ols were more active than coumarins, with (‒)-epicatechin reaching up to 80% inhibition. Esculin was characterized by the lowest protective activity. The effect for this compound was significant from the concentration of 5 µg/mL and reached, maximally (at 50 µg/mL), about 35% (Figure 4).

### 3.5. Influence on Plasma Hemostasis Parameters

**Figure 5:**
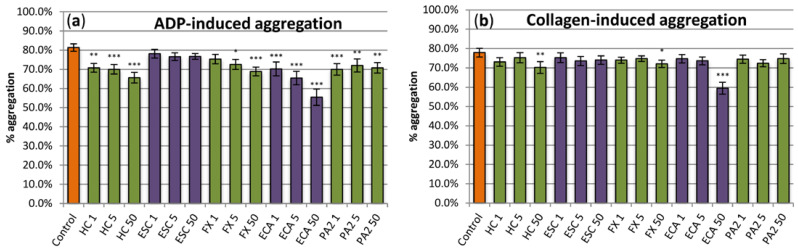
Evaluation of the anti-platelet effects of the examined analytes. The hemostatic response of blood platelets (aggregation in the platelet-rich plasma; PRP) was induced by (**a**) ADP and (**b**) collagen. Results presented as means ± SE (*n* = 11). Statistical differences: * *p* < 0.05, ** *p* < 0.01, *** *p* < 0.001 for control platelets versus platelets incubated with analytes. In PRP samples treated with a reference drug, i.e., the aspirin DL-lysine (Lys-ASA; 200 µg/mL), the ADP- and collagen-induced blood, platelet aggregation was inhibited by 100% (*n* = 3). Analytes: HC, horse-chestnut bark extract; ESC, esculin; FX, fraxin, ECA, (‒)-epicatechin; PA2, procyanidin A2; the numbers after analyte symbol indicate concentration in μg/mL.

Analyses of the anti-thrombotic potential of the examined extract included two aspects, i.e., evaluation of its anti-platelet action (1) and assessment of its ability to reduce blood clotting (2). At concentrations of 1–50 μg/mL, the extract exhibited slight (up to about 18%) but statistically significant inhibitory effect on platelet aggregation induced by ADP (Figure 5a). On the other hand, the statistically significant effect on platelet aggregation induced by collagen was found only at the highest (50 µg/mL) concentration of the extract (Figure 5b). Among constituents, flawan-3-ols exhibited stronger effects on ADP-induced aggregation than coumarins. (‒)-Epicatechin was visibly the strongest inhibitor, diminishing the aggregation by up to about 30% at the concentration of 50 µg/mL. On the other hand, from the two tested coumarins, only fraxin inhibited the ADP-induced aggregation, at concentrations of 5–50 μg/mL (Figure 5a). In the case of the collagen-induced aggregation, the statistically significant effects for constituents were only observed for (‒)-epicatechin and fraxin at the highest (50 µg/mL) level (Figure 5b).

**Figure 6:**
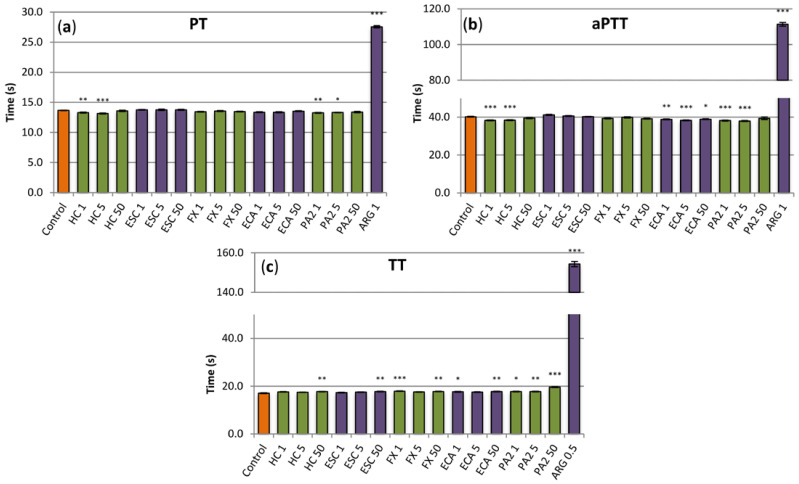
Effects of the investigated analytes on coagulation times, (**a**) prothrombin time (PT), (**b**), activated thromboplastin time (aPTT) and (**c**) thrombin time (TT). Results presented as means ± SE (*n* = 10–12). Statistical differences: * *p* < 0.05, ** *p* < 0.01, *** *p* < 0.001 for control plasma versus plasma incubated with analytes. Analytes: HC, horse-chestnut bark extract; ESC, esculin; FX, fraxin, ECA, (‒)-epicatechin; PA2, procyanidin A2; ARG, argatroban; the numbers after analyte symbol indicate concentration in μg/mL.

The examined substances displayed weak anticoagulant activity; however, in some cases, the observed effects were statistically significant (Figure 6). In low concentrations (1 µg/mL and 5 µg/mL), the extract slightly decreased PT and aPTT (Figure 6a,b), while, at the highest concentration, it increased TT but only marginally (Figure 6c). Some tendency to increase TT was also visible for all of the tested extract constituents, while flavan-3-ols, especially procyanidin A2, decreased minimally PT and/or aPTT (Figure 6). This latter effect, similarly as in the case of the extract, was also noticeable at low concentrations of both constituents. Nevertheless, all effects observed for the extract and its constituents were negligible, when compared to a reference anticoagulant drug, i.e., argatroban (Figure 6).

## 4. Discussion

The main area of application of horse-chestnut bark is conditions connected with vascular damage, such as varicose veins or hemorrhoids [2]. While the etiology of those diseases has not been yet fully explained, oxidative stress is one of the mechanisms implied in the process [3]. Reactive oxygen species (ROS) play an important role in vascular signaling, but their excess may have a detrimental impact on vascular health. For example, O_2_^•−^ is required for cell growth, but, when overproduced, it reacts with NO^•^, promoting vasoconstriction and endothelial dysfunction; furthermore, the ONOO^−^ that forms in that reaction is one of the most destructive species [23]. Similarly, physiologically produced H_2_O_2_ modulates endothelial cell function, such as vasorelaxation or cell proliferation; however, in supraphysiological levels, it may impair proliferation or induce hypertrophy of vessel muscles [24]. Undergoing inflammatory processes in chronic venous insufficiency may promote oxidative stress and, indeed, it has been shown that the generation of ROS in insufficient veins is increased and may further induce the progress of the disease [25,26]. Direct scavenging of ROS is one of the most basic mechanisms in which the polyphenols present in plant extracts may be helpful in alleviating oxidative stress disorders [3]. In previous studies, the bark of *A. hippocastanum* has been shown to be a more efficient scavenger of DPPH radical than other parts of the tree (flowers, leaves, or seeds) [7]. It has also been proved effective in scavenging O_2_^•−^ and HO^•^ in an in vitro EPR study [8]. The results of the present experiments confirm the findings of Braga et al. [8]; additionally, the wider spectrum of the investigated ROS and use of a universal standard (ascorbic acid) allow for additional conclusions. With regard to the ascorbic acid, a ubiquitous endogenous antioxidant, the reactivity of the bark extracts is pronounced the most towards HO^•^, O_2_^•−^ and ONOO^−^. On the other hand, the bark performs much worse than AA against NO^•^. Such activity profile might be to some extent beneficial, as NO has a mainly positive function in the vascular system (e.g., vessel dilatation or promotion of fibrinolysis) and its negative effects are mostly due to the ONOO^−^ arising in the reaction of NO^•^ and O_2_^•^^−^ [27]. Previous works attributed the extract antioxidant capacity to polyphenols in general [7,8], but no investigation into the activity of the individual compounds has been executed. Present results clearly show that, while all of the main constituents have concentration-dependent scavenging potential, the flavan-3-ol derivatives are much more efficient than coumarins.

In vitro chemical scavenging assays might serve only as a preliminary assessment of the antioxidant capacity. Thus, in the further steps of our investigation, more relevant biological analyses were performed to evaluate the potential benefits of the extract and its constituents for the human organism. The analytes were tested in the concentration range of 1–50 µg/mL, which, in the case of the extract, is an equivalent of about 0.4–20 µg of the phenolics/mL. The lower levels of that range were selected to correspond to the levels of coumarins and flavan-3-ols present in plasma after oral administration, which, depending on the initial dose and form of the preparation, might reach about 0.35–1.7 µg/mL [28,29]. On the other hand, the upper level of the concentration range was selected to reflect higher amounts of analytes that might be locally available during topical application, although it was limited by the solubility of the analytes under experimental conditions.

When fighting ROS effects, the organism has its own defense system that includes, among others, enzymatic and non-enzymatic antioxidants. While available research indicates that the activity of antioxidant enzymes, such as SOD, in varicose veins is up-regulated probably as a response to increased O_2_^•−^ production [25,26,30,31], the NEAC of the plasma might be depleted by a higher demand [25,31]. A significant decrease in the total thiol level in plasma in patients with varicose veins in comparison to control subjects was also recorded, including the depletion of reduced glutathione that constitutes an important part of the non-enzymatic antioxidant system [32]. Our results show, for the first time, that the extract from horse-chestnut bark has the capacity to restore and/or improve the NEAC of plasma. Moreover, the extract, in concentrations as small as 1 μg/mL, had also a protective effect on protein thiol levels in ONOO^−^ treated plasma. The differences between particular constituents are less pronounced than in the scavenging assays. That may be caused by a higher complexity of the model and indicates that some additional interactions come in the way (e.g., protein binding, etc.). However, the weaker activity of the coumarins, especially esculin, might still be inferred. The correlation between the compounds scavenging potential and their efficacy in maintaining NEAC and thiol levels may imply that direct quenching of ONOO^−^ and derived radicals plays an important role in those effects.

The negative ROS effects in vivo include, among others, the disruption of main biomolecules, such as lipids and proteins [27]. Lipids present in biomembranes undergo peroxidation that may lead to the destruction of cell walls and organelles. Proteins, on the other hand, might be nitrated by nitrogen-containing ROS (also known as RNS, reactive nitrogen species, e.g., ONOO^−^, NO^•^) and either change or totally lose their function [10]. The destructive action of ROS may be traced by assessing the levels of specific markers. For example, lipid peroxidation is evidenced by increased amounts of the end products of the process, malonylodialdehyde (MDA) and its derivatives, that are often also described as TBARS, due to their reactivity with thiobarbituric acid. Increased levels of MDA have been found in patients with varicose veins [33] and more detailed studies have linked the increase in the levels of that marker with thrombophlebitis [34] and venous ulcers [30]. Levels of 3-NT serve as indicator of protein nitration. Some of the available data show a strong correlation between the levels of that marker and the severity of the venous disorders [35]. Elevated 3-NT is indeed connected with endothelial dysfunction and also indicated as a risk factor in many other cardiovascular complaints [36]. Extract from horse-chestnut bark has been proven before to inhibit peroxidation of lipids in liver microsomes obtained from sheep [7]. In the present paper, we showed that it also protects the lipids of human plasma. Moreover, for the first time, we demonstrated a strong dose-dependent capacity to prevent protein nitration induced by ONOO^−^. Those results suggest that the extract might alleviate the negative effects of ROS and hinder the progression of the disease caused by oxidative stress.

Contrary to the other plasma assays, in the 3-NT evaluation, (‒)-epicatechin and both coumarins seem to have similar potential, somewhat superior to that of procyanidin A2 and antioxidant standard AA. This fact would suggest that some mechanisms, other than direct ROS quenching, might be important for that effect. Those mechanisms might include, e.g., competition with tyrosine for nitration, which has previously been suggested for (‒)-epicatechin and other flavonoids [37].

The oxidative/nitrative damage caused by superfluous ROS to macromolecules might well concern proteins involved in the coagulation cascade. The results of recent investigations highlight, e.g., the role of nitrated fibrinogen in the potential development of coagulation disorders [38]. For example, the elevated levels of nitrated fibrinogen have been found in patients with venous thromboembolism and seem to reflect an ongoing oxidative and inflammatory state of the venous system [38]. Conversion of fibrinogen to fibrin is the last step of clot formation and nitrative modification of fibrinogen have been found to lead to irregularities both in the course of the process and in the structure and properties of the resulting clot [39,40]. It has been established that even very low nitration of fibrinogen molecules (~45–65 μmol nitrotyrosine/mol tyrosine in this protein) modifies the hemostatic properties of fibrinogen, leading to an increased fibrin generation and changes in the clot architecture [40]. As our study showed, the horse-chestnut extract and its main constituents are able to protect fibrinogen from nitrative damage caused by ONOO^−^ and, thus, they may partially hinder the abnormal clotting patterns. The flavan-3-ols derivatives were more potent than coumarins, which is consistent with their higher scavenging potential. The high activity of (‒)-epicatechin is especially in accordance with the previous results obtained for this compound, which further showed that, through its protective anti-nitration potential, (‒)-epicatechin is indeed able to diminish the nitration-induced pro-thrombotic impact on fibrinogen clotting behavior [10].

To diminish the increased risk of clot formation, that, in venous insufficiency, might also be a result of the slower blood flow and endothelial injury, anti-aggregatory or anticoagulant drugs are often used [14,41]. Plant extracts might have similar effects, which, on one hand, might constitute one of the mechanisms of their activity and, on the other, might cause interactions with already taken medications [42]. Thus, it is important to study and recognize those effects. In the present study, we established that the extracts and its constituents hindered the ADP-induced platelet aggregation, while having hardly any effect on the aggregation induced by collagen. Although statistically significant, the effects were, at most, moderate. Thus, they might be considered as an auxiliary mechanism of action only. For further research, it might be interesting to check if this effect is synergistic with the effect of known anti-aggregatory drugs, such as pentoxifylline or calcium dobesilate, often taken by patients with symptoms of venous insufficiency [41]. Similarly as in the case of scavenging activity, among the individual constituents, flavan-3-ols were more effective than coumarins, which further supports their role as important active ingredients of the bark. (‒)-epicatechin, that was the most efficient analyte in our study, has previously been found to be a strong inhibitor of ADP-induced platelet aggregation; an interference with the P2Y12 receptor was suggested, but further studies are required to confirm the exact mechanism of action [13].

Coumarins and coumarin-rich plant substances are often linked with anti-coagulant properties [3,12]. However, as our results show, the impact of the horse-chestnut extract and its constituents on clotting times in vitro might not be considered as clinically relevant. The coumarins themselves weakly increased TT, but neither esculin nor fraxin possess the structural features of strong coumarin anticoagulants, i.e., hydroxyl group at C-4 [43]. The results for flavan-3-ols were inconclusive with very weak pro- and anti-coagulant effects, depending on the concentration. Previous studies on (‒)-epicatechin showed indeed no influence on clotting times [44,45]. On the other hand, (‒)-epicatechin was found to weakly inhibit the amidolytic activity of thrombin [46] and had a moderate anticoagulant activity in global plasma coagulation tests [13]. The reason for those discrepancies might be a very short experiment time of in vitro clotting time assays, that allows to identify only the strongest effects. Thus, a weaker anticoagulant capacity of the investigated extract and its constituents cannot be excluded and more detailed investigations are required to clarify this issue.

## 5. Conclusions

In the present study, the antioxidant, anti-platelet and anticoagulant activities of horse-chestnut bark extract and its constituents were studied. The extract possesses relevant scavenging activity towards the most common in vivo oxidants and has been proved to protect the plasma lipids and proteins against the changes induced by ONOO^−^. The latter activity is particularly manifested in preventing the oxidative damage of fibrinogen. Thus, it was shown that antioxidant capacity might be one of the important mechanisms of the horse-chestnut bark efficacy in the vascular conditions. On the other hand, the anti-aggregatory activity of the extract was only moderate, but might still be considered as an auxiliary mechanism, or in the context of possible synergistic effects with other drugs. In addition, more studies are required to fully explain the impact on clotting times; however, those effects do not seem to have applicatory influence on the overall efficiency and safety of the plant material. With regard to the contribution of the individual constituents, our results indicate that, considering their total content and activity parameters, flavan-3-ols should be considered as the main vectors of the investigated activities with supporting role of the coumarin glycosides.

## Figures and Tables

**Table 1:** Content of individual constituents in the dry methanol extract from the bark of *A. hippocastanum*.

No.	Analyte	Content (mg/g)
1	Esculin	175.23 ± 2.32
2	Isoscopolin	3.63 ± 0.16
3	Scopolin	2.83 ± 0.07
4	Esculetin	10.03 ± 0.10
5	Fraxin	73.69 ± 0.99
	Total coumarins	265.42 ± 3.30
6	(‒)-Epicatechin	63.17 ± 0.73
7	Proanthocyanidin dimer B-type	14.14 ± 0.77
8	Proanthocyanidin trimer A-type	5.34 ± 0.72
9	Procyanidin A2	55.91 ± 0.35
	Total flavan-3-ols	138.56 ± 2.38
	Total phenolics	403.98 ± 3.86

Identification and quantification according to the method described previously [5]. Data presented as means ± SE (*n* = 3).

**Table 2:** Scavenging activity of the dry methanol extract from the bark of *A. hippocastanum* and its main constituents towards selected reactive oxygen species.

Analyte	SC_50_ (µg/mL)				
	H_2_O_2_	OH^•^	O_2_^•−^	NO^•^	ONOO^−^
*A. hippocastanum* extract	38.03 ± 1.74 ^C^	172.27 ± 4.57 ^C^	9.96 ± 0.90 ^B^	8.74 ± 0.35 ^D^	140.44 ± 2.37 ^D^
Esculin	574.43 ± 23.63 ^E^	>1000	69.27 ± 5.35 ^C^	8.56 ± 0.10 ^D^	>1200
Fraxin	124.71 ± 2.75 ^D^	>1000	358.06 ± 31.37 ^D^	12.90 ± 0.21 ^E^	>200
(–)-Epicatechin	8.41 ± 0.30 ^A^	99.53 ± 0.85 ^A^	7.89 ± 0.99 ^AB^	0.91 ± 0.04 ^B^	35.70 ± 2.32 ^A^
Procyanidin A2	11.23 ± 0.68 ^B^	97.26 ± 1.74 ^A^	6.33 ± 0.68 ^A^	1.75 ± 0.02 ^C^	44.61 ± 1.19 ^B^
Ascorbic acid	11.71 = 0.76 ^B^	152.59 ± 2.79 ^B^	6.13 ± 0.67 ^A^	0.48 ± 0.03 ^A^	83.34 ± 1.50 ^C^

Data presented as means ± SE (*n* = 5). In each column values labeled with the same capital (A–E) are not statistically different at *α* = 0.05.

## Data Availability

Data is contained within the article.
